# A discrete-to-continuum model of protein complexes

**DOI:** 10.1007/s10237-022-01564-7

**Published:** 2022-03-25

**Authors:** Paolo Maria Mariano, Marco Bacci

**Affiliations:** 1 DICEA, Università di Firenze, via Santa Marta 3, I-50139 Firenze, Italy; 2 Department System Analysis, Integrated Assessment and Modelling, EAWAG, Überlandstrasse 133, Ch-8600 Düberdorf, Switzerland

**Keywords:** Protein dynamics, Discrete-to-continuum schemes, Multi-field theories

## Abstract

On the basis of a tensor representation of protein shape, obtained by an affine decomposition of residue velocity, we show how to identify actions at continuum scale for both single proteins and their complexes in terms of power equivalence. The approach constructs and justifies a continuum modeling of protein complexes, which avoids a direct, atomistic-based, simulation of the whole complex, rather it focuses (in a statistical sense) on a single protein and its interactions with the neighbors. In the resulting setting we also prove the existence of equilibrium configurations (native states) under large strains.

## Introduction

Protein complexes are a result of the binding among proteins or protein and lingands. The knowledge of their structure and behavior plays an essential role in drug design (see, e.g., B-Rao et al. (2009), Meng et al. (2011), Naqvi et al. (2018), Gouaux (1998)).

For them we build up a discrete-to-continuum model on the basis of a local affine approximation for the motion of each single protein. Our approach allows us to retain the main features of a fully direct simulation while it reduces the computational burden because just atomistic-based computations for a single protein and the links with its neighbors are necessary. The resulting continuum scheme that we propose falls within the general model-building framework for the mechanics of complex materials (Capriz 1989; Mariano 2002, 2014).

Building up on the conceptual structures of that framework, we identify gross-scale and microstructural stresses in terms of the atomistic interactions within and among proteins. We consider them to be of beam-like type. Then, the pertinent potential $V_{tot}$ can be chosen to be

$$\begin{aligned} \begin{aligned} V_{tot}(r, \theta , \phi ) =&\sum _{i=1}^{N-1}\frac{k_h}{2}(r_{i,i+1} - R_{i,i+1})^2 + \sum _{i=2}^{N-1} \frac{k_{\theta }}{2}(\theta _{i} - \Theta _{i})^{2} \\&+\sum _{i=3}^{N-2} k_{\phi }^{(1)}[1 - \cos (\phi _{i} - \Phi _{i})] \\&+ k_{\phi }^{(3)}[1 - \cos 3(\phi _{i} - \Phi _{i})] +\sum _{i,j>i+3} V_{nb}(r_{ij}), \end{aligned} \end{aligned}$$

where *N* is the total number of amino acids, $r_{ij}$ the distance between residues *i* and *j*, measured from one backbone atom (C$_{\alpha }$) to the other, $\theta _{i}$ the bending angle identified by three consecutive C$_{\alpha }$s (namely $i-1$, *i*, $i+1$), $\phi _{i}$ the torsional angle referred to the two planes identified by four consecutive C$_{\alpha }$s (i.e., $i-2$, $i-1$, *i*, $i+1$). $R_{ij},\Theta _{i},$ and $\Phi _{i}$ are ground-state values of $V_{tot}$ and are set by the crystallographic structure of the protein. Such a choice allows one to describe folding, which influences translocation through tissue pores and the way proteins interact with each other forming complexes.

The non-bound term

$$\begin{aligned} V_{nb}(r_{ij}) = \epsilon \left\{ \begin{array}{lc} \left[ 5 \left( \frac{R_{ij}}{r_{ij}} \right) ^{12} - 6 \left( \frac{R_{ij}}{r_{ij}} \right) ^{10} \right] &{} \text {for} \;\;R_{ij} < R_c\\ \frac{10}{3} \bigg (\frac{\sigma }{r_{ij}} \bigg )^{12} &{} \text {for} \;\;R_{ij} > R_c, \end{array} \right. \end{aligned}$$

where $\epsilon$ indicates the energy scale and $R_c$ is the so-called cutoff radius in the Gō-like approach, accounts for non-local interactions ($|j-i| >3$) along the sequence. $R_c$ selects the number of native contacts and controls stability. The larger $R_c$ the more stable is the native structure.

**Fig. 1:**
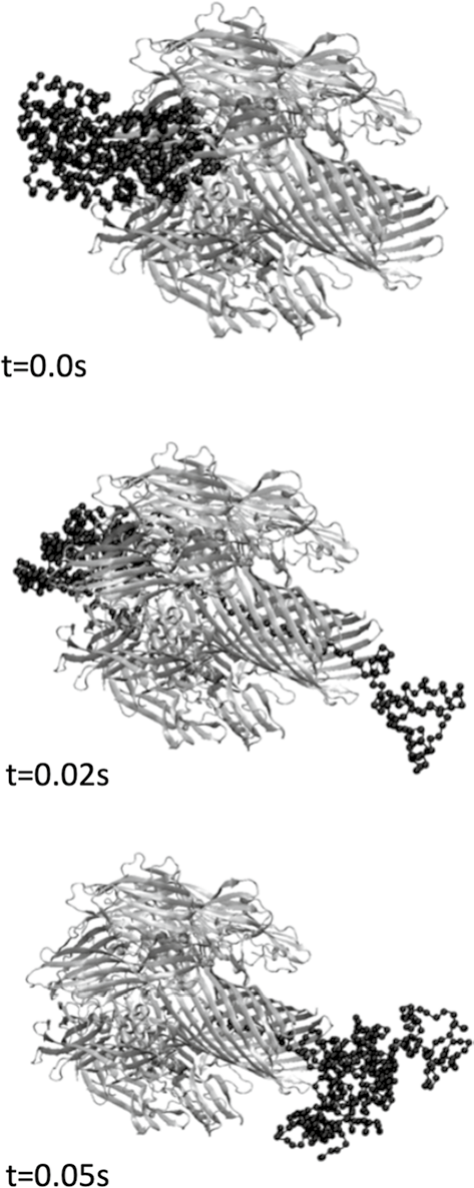
Snap-shot of a translocation across a pore, computed taking into account the discrete structure of the Maltose Binding Protein

**Fig. 2:**
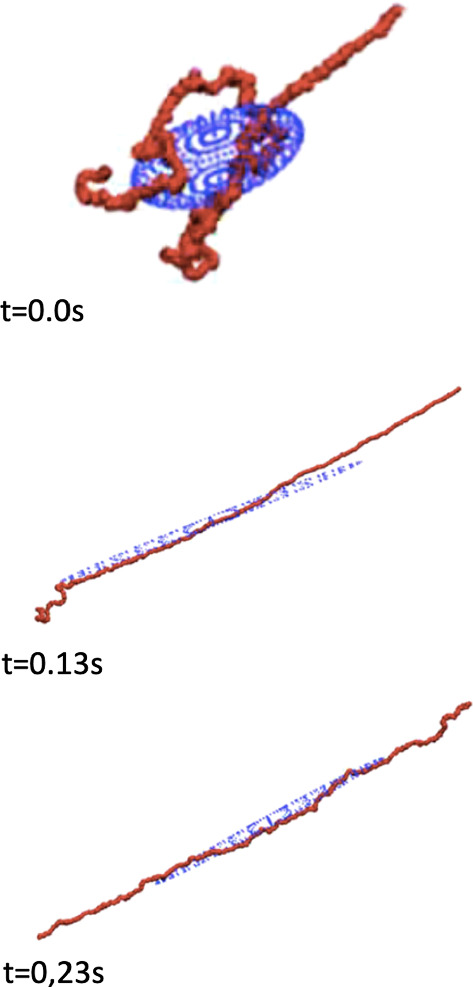
Tensor approximation of a protein de-folding process: the ellipsoid represent the second-rank tensor $\nu$

Both $R_c$ and $\epsilon$ establish the denaturation temperature $T_f$. In the simulations leading to Figs. 1 and 2, which are connected with the computational analyses performed in references (Bacci and Mariano 2021) and (Bacci and Mariano 2014), we refer to the values $\epsilon = 1$, $R_c = 3.0 \mathring{A}$ and $R_c = 7.5 \mathring{A}$. Non-adjacent residues *i* and *j* attract each other when a *native contact* is established between the two, i.e., their distance is lower than $R_c$. Alternatively, they interact through a soft repulsive potential with core $\sigma = 4.5 \mathring{A}$. The dynamics of a single amino acid can be viewed as ruled by

$$\begin{aligned} m\ddot{\mathbf {r}}_i = - \gamma {\mathbf {\dot{r}}_i(t)} - \frac{\partial {V(\mathbf {r})}}{\partial \mathbf {r}_i}|_{\mathbf {r}_i} + \Gamma (t), \end{aligned}$$

in the over-damping limit, where ${\mathbf {r}}_i$ is the position vector of the $i$th residue with mass *m* (all amino acids assumed with the same mass), $V(\mathbf {r})$ the total potential acting on it (which includes $V_{tot}$ and the one associated with the environmental action), $\Gamma (t)$ a Gaussian white noise vector contribution, and $\gamma$ a friction coefficient. *V* depends on the position vectors of all amino acids constituting the protein. As usual, the superposed dot indicates time derivative.

Even just for a single protein, the direct simulation of its dynamics performed by taking into account all protein residues, as in the scheme sketched above, is computationally hard, due to the high number of residues constituting each protein. The computational burden increases drastically when we deal with protein complexes. So, we are pushed to consider a coarse grained continuum approach. Local atomistic simulations pave the way to field-based computations of the whole protein complex, along a path pertaining to multi-scale simulation methods (Liu et al. 2006).

For the coarse-grained description of a single protein translocation, common proposals rest on a representation of the molecule in terms of a scalar, which is transported in free space or in a constrained environment, e.g., across a tissue (Krivov and Karplus 2004; Makarov 2008; Makarov et al. 2005; Lu and Schulten 1999).

A refined approach adopts a second-rank tensor, say $\nu \in \mathbb{R}^{3}\otimes \mathbb{R}^{3}$, which allows a more refined description of protein changes in shape, a choice that we adopt here. So, as proposed in references (Bacci and Mariano 2014) and (Bacci and Mariano 2021), we decompose the dynamics of a protein into two factors: mass center motion and relative changes in shape.

Questions emerge:

- Which way do we identify $\nu$?
- How can we summarize inter-amino-acid interactions and environmental ones in order to describe their influence on the dynamics of protein mass center and the affine fluctuations around it described by $\nu$?
- Which conditions assure existence of equilibrium states under large strains?

We tackle here these questions, formulating possible answers. The scheme we propose offers a way to develop field-based large-scale simulations for the mechanics of protein complexes.

*Notations*. In what follows $\mathrm {Hom}(A, B)$ will indicate the space of linear maps between the linear spaces *A* and *B*, specified every time. An interposed dot between, say, *C* and *D*, namely $C\cdot D$, will denote duality pairing, between an element of a linear space and one of its dual, a pairing identified with the standard scalar product when the pertinent metrics are flat.

## Tensor representation of a single protein

### Local affine approximation of a single protein motion: consequences and limits

Let $\mathbf {w}_i$ be a velocity of the *i*-th amino acid. We take for it the decomposition

1 $$\begin{aligned} \mathbf {w}_i(\mathbf {r}_{i},x,t)=\mathrm {v}(x,t) + \dot{\nu }(x,t)\mathbf {r}_{i} + c_{i}(\mathbf {r}_{i},x,t), \end{aligned}$$

where $\mathrm {v}(x,t)$ is a velocity of the protein mass center, placed in the instant *t* at *x*, a point to which we refer the amino acid position vector $\mathbf {r}_{i}$. The product $\dot{\nu }\mathbf {r}_{i}$ reads in components as $\dot{\nu }^{h}_{\;k}\mathbf {r}_{i}^{k}$, with summation assumed over repeated indices. We will maintain this compact notation throughout this paper.

We choose $\nu$ to be such that $\mathrm {v} + \dot{\nu }\mathbf {r}_{i}$ best fits the overall protein kinetic energy; in other words, with *N* the number of amino acids in the protein, $\dot{\nu }$ is an argument minimizing the difference

$$\begin{aligned} \sum _{i=1}^{N}|\mathbf {w}_i-(\mathrm {v} + \dot{\nu }\mathbf {r}_{i})|^{2}, \end{aligned}$$

where, as usual, the vertical bars indicate modulus. This last choice for $\nu$ recalls one in the discrete-to-continuum description of sparse phases (Capriz 2008), when we homogenize at continuum scale the fluctuating motion of a cluster of disconnected grains (Capriz and Mariano 2014; Capriz and Giovine 2017; Capriz and Mariano 2018).

Tensor $\nu$ enters the mechanical description of protein motion, while thermal effects are referred to *c*, precisely to the kinetic energy associated with *c*, or its tensor counterpart, which involves the dyad $c_{i}\otimes c_{i}$. When we refer to the protein mass center, and neglect *c* at first glance, the affine approximation $\dot{\nu }\mathbf {r}_{i}$ is, indeed, a version of the Cauchy-Born rule (commonly discussed with reference to crystals E W. Lu 2013) here written in terms of time rates rather than placements (for this traditional version see also Ericksen (2008)).

By looking at a single protein in an given environment, we consider two distinct balances of actions. One refers to mass center motion, and we write for it

2 $$\begin{aligned} \mathbf {f}^{\ddagger }=0 \end{aligned}$$

assuming the decomposition

$$\begin{aligned} \mathbf {f}^{\ddagger }=\mathbf {f}+\mathbf {f}^{in}\;, \end{aligned}$$

with $\mathbf {f}$ a non-inertial force to be identified at this stage and $\mathbf {f}^{in}$ the inertial action, which we identify with $-m_{p}\dot{\mathrm {v}}$, where $m_{p}$ is the protein total mass, by imposing that the power of $\mathbf {f}^{in}$ is balanced by the kinetic energy time variation, namely $\mathbf {f}^{in}\cdot \mathrm {v}=-\frac{\mathrm {d}}{\mathrm {d}t}\big (\frac{1}{2}m_{p}|\mathrm {v}|^{2}\big )$, for any choice of the velocity. Eventually, the balance equation for the mass center motion is the standard Newton law

3 $$\begin{aligned} m_{p}\dot{\mathrm {v}}=\mathbf {f}. \end{aligned}$$

The other balance is the one of actions performing power in the affine motion relative to the mass center, the one with time rate $\dot{\nu }$. For it we formally write

$$\begin{aligned} \beta =z\;, \end{aligned}$$

where $\beta$ is a second-rank tensor describing the environmental actions over the protein, while *z*, also a second-rank tensor, summarizes amino-acid-to-amino-acid actions inside the protein, both tensors to be identified at this stage in terms of interactions at the protein discrete scale. We presume that *z* admits the additive decomposition

$$\begin{aligned} z=z^{d}+z^{e}, \end{aligned}$$

with $z^{d}$ an intrinsically dissipative component in the sense that we presume $z^{d}$ to satisfy the mechanical dissipation inequality

$$\begin{aligned} z^{d}\cdot \dot{\nu }\ge 0 \end{aligned}$$

for every choice of time rate $\dot{\nu }$, the equality sign holding true if and only if $\dot{\nu }=0$. An admissible expression of $z^{d}$ for the previous inequality is

$$\begin{aligned} z^{d}=a\dot{\nu }, \end{aligned}$$

with *a* the value of a positive-definite real-valued state function $\tilde{a}(\dots )$ of space and time. Consequently, the balance $\beta =z$ becomes $\beta -z^{e}=a\dot{\nu }$. Taking $\tilde{a}(\dots )$ to be a positive constant, we rewrite the previous balance as

4 $$\begin{aligned} \dot{\nu } = z_{s}, \end{aligned}$$

where $z_{s}$ indicates $\beta -z^{e}$ divided by *a*. This is the evolution equation for the around-protein-mass-center affine motion. In fact, $\beta$ can include inertial effects associated with affine vibrations. However, we disregard here relative inertia associated with the protein around-the-mass-center motion.

With $\mathbf {f}_{i}$ the non-inertial environmental force exerted over the $i-$th amino acid and $\mathbf {h}_{ij}$ the one exerted over the same amino acid by the $j-$th one, we write the power $\mathcal {P}$  that they perform over the whole protein as

5 $$\begin{aligned} \mathcal {P}:=\sum _{i=1}^{N} \mathbf {f}_{i}\cdot \dot{\mathbf {r}}_i+\sum _{i=1}^{N}\sum _{j\in Cont(i)}\mathbf {h}_{ij}\cdot \dot{\mathbf {r}}_i, \end{aligned}$$

where *Cont*(*i*) is the set indexing the amino acids that share bonds with the $i-$th one. In the definition (5) we take $\mathbf {h}_{ij}=\mathbf {h}_{ji}$. By using the decomposition (1), we then get

6 $$\begin{aligned} \begin{aligned} \mathcal {P}:=\bigg (\sum _{i=1}^{N} \mathbf {f}_{i}+\sum _{i=1}^{N}\sum _{j\in Cont(i)}\mathbf {h}_{ij}\bigg )\cdot \mathrm {v}&+\bigg (\sum _{i=1}^{N} \mathbf {f}_{i}\otimes \mathbf {r}_i+\sum _{i=1}^{N}\sum _{j\in Cont(i)}\mathbf {h}_{ij}\otimes \mathbf {r}_i\bigg )\cdot \dot{\nu }\\&+\bigg (\sum _{i=1}^{N} \mathbf {f}_{i}\cdot c_{i}+\sum _{i=1}^{N}\sum _{j\in Cont(i)}\mathbf {h}_{ij}\cdot c_{i}\bigg )\;, \end{aligned} \end{aligned}$$

where, as usual, the symbol $\otimes$ indicates *tensor product*. We write $\mathcal {P}^{prot-fluc}$ for the fluctuation component of $\mathcal {P}$, given by

$$\begin{aligned} \mathcal {P}^{prot-fluc}:=\bigg (\sum _{i=1}^{N} \mathbf {f}_{i}\cdot c_{i}+\sum _{i=1}^{N}\sum _{j\in Cont(i)}\mathbf {h}_{ij}\cdot c_{i}\bigg )\;. \end{aligned}$$

As a basic identification relation we impose the identity

7 $$\begin{aligned} |\hat{\mathbf {e}}|^{-1}(\mathcal {P}-\mathcal {P}^{prot-fluc})=\mathbf {f}\cdot \mathrm {v}+z_{s}\cdot \dot{\nu }, \end{aligned}$$

where $|\hat{\mathbf {e}}|$ is the volume of the protein crystallized structure. We presume that the identity is valid for every choice of $\mathrm {v}$ and $\dot{\nu }$. The arbitrariness of these rates implies

8 $$\begin{aligned} \mathbf {f}&=|\hat{\mathbf {e}}|^{-1}\bigg (\sum _{i=1}^{N} \mathbf {f}_{i}+\sum _{i=1}^{N}\sum _{j\in Cont(i)}\mathbf {h}_{ij}\bigg ), \end{aligned}$$

9 $$\begin{aligned} z_{s}&=|\hat{\mathbf {e}}|^{-1}\bigg (\sum _{i=1}^{N} \mathbf {f}_{i}\otimes \mathbf {r}_i+\sum _{i=1}^{N}\sum _{j\in Cont(i)}\mathbf {h}_{ij}\otimes \mathbf {r}_i\bigg )\;. \end{aligned}$$

The first addendum of the right-hand-side term is associated with $\beta$, because it is an external action, while the second gives us $-z^{e}$. When we do not consider external forces, $z_{s}$ reduces to

$$\begin{aligned} \sum _{i=1}^{N}\sum _{j\in Cont(i)}\mathbf {h}_{ij}\otimes \mathbf {r}_i. \end{aligned}$$

This scheme is at the basis of extended numerical simulations focusing of a Maltose Binding Protein, a cluster of 370 amino acids connected each other by the potential (1), performed in references (Bacci and Mariano 2021) and (Bacci and Mariano 2014) at temperature $T = 0.75 \hat{\theta}$ (in the so-called *code measure units*) by neglecting the gross motion, i.e., equation (3), and considering across-pore-translocation under a channel potential

$$\begin{aligned} V_{pore}(x_{1},x_{2},x_{3}) = V_0\left( \frac{x_{2}^2+x_{3}^2}{R_p^2} \right) ^q \tilde{\Theta }[x_{1} (L-x_{1})], \end{aligned}$$

where $\tilde{\Theta }(s) = [1 + \tanh (\alpha s)]/2$ is a smooth step-like function limiting the action of pore potential in the region [0, *L*], with pore length *L* varying from $100 \mathring{A}$ to $800 \mathring{A}$, and $R_p=10 \mathring{A}$ as indicated by $\alpha$HL structural data (Gouaux 1998). Commonly, a convenient choice of the other parameters is $q=1$, $\alpha =3 \mathring{A}^{-2}$ and $V_0 = 2\epsilon$. In the simulations leading to Figs. 1 and 2, a driving force $\mathbf {f}$ acts along the pore axis only in the capture region $[-2,0]$ and inside the pore [0, *L*], by dragging the foremost protein residue. We computed the eigenvalues—say $\lambda _{i}$, with $i=1,2,3$—of the symmetric factor *U* in the polar decomposition $\nu =RU$, with $R\in O(3)$ and *U* a second-rank symmetric tensor ($U\in \mathrm {Sym}(\mathbb {R}^{3},\mathbb {R}^{3})$), considering their square roots as the semi-axes of an ellipsoid (see Fig. 2). Comparison of AFM-like, stretching, and translocation results with the edge evolution of a smallest box bounding the protein show that the identification (9), based on the affine-type decomposition (1), appears appropriate above all in confined dynamics, when fluctuations are bounded, as shown in references (Bacci and Mariano 2021) and (Bacci and Mariano 2014).

Further results of the extended numerical campaign we realized for a single protein are in reference (Bacci and Mariano 2021).

### Overcoming limits of the previous formulation

A way to overcome the mentioned limits, due to thermalization-induced non-homogeneous deformations, is to assume that $z_{s}$ is such that the second sum in (9) is replaced by

10 $$\begin{aligned} \sum _{i=1}^{N}\sum _{j\in Cont(i)} k^{z_2}_{j} \mathbf {h}_{ij}^{+}\otimes \partial _{t}\mathbf {r}^{G+}_{j}, \end{aligned}$$

where $\partial _{t}$ indicates partial time derivative; also $\mathbf {h}_{ij}^{+}$ and $\mathbf {r}^{G+}_{j}$ are obtained from $\mathbf {h}_{ij}$ and $\mathbf {r}^{G}_{j}$ by taking the absolute values of their components. Moreover, $k^{z_2}_{j}$ is a scalar defined by

$$\begin{aligned} k^{z_2}_{j} = r_{g_{0}}^{-1}|\mathbf {r}^{G}_{j}|, \end{aligned}$$

with $r_{g_{0}}$ the gyration radius of the protein crystallized structure. The choice of $\mathbf {h}_{ij}^{+}$ and $\mathbf {r}^{G+}_{j}$ is a projection of protein dynamics onto the first octant of a frame chosen in $\mathbb {R}^3$. The vector $\mathbf {h}_{ij}$ is the algebraic sum of elementary actions in the Gō-like scheme, those stemming from peptide bonds and bending effects. This choice is computationally inexpensive and describes appropriately free motion and protein translocation through a pore. However, if during this last process the pulling stalls, the resulting $\nu$ and its symmetric component *U* do not correctly reproduce the process itself (see remarks in reference Bacci and Mariano 2021), while in the remaining cases, the variation of $\nu$ in time closely describes the protein evolution. An alternative choice for $z_{s}$ is to replace (10) with

11 $$\begin{aligned} \sum _{i=1}^{N}\sum _{j\in Cont(i)}k^{z_3}_{ij}(\mathbf {h}_{ij}\cdot \mathbf {l}_{ij}\mathbf ){\varvec{\iota }} \otimes \partial _t\mathbf {r}^{G+}_{j}, \end{aligned}$$

where $\mathbf {l}_{ij}$ is a dimensionless normalized vector indicating the direction through *i*-th and *j*-th residues. $k^{z_3}_{ij}$ is a dimensionless factor defined by

$$\begin{aligned} k^{z_3}_{ij}:=\mathrm {sign}(| \mathbf {r}_{ij}|-| \mathbf {r}_{ij_{0}}|)r_{g_{0}}^{-1}| \mathbf {r}^G_{j}|, \end{aligned}$$

where $\mathbf {r}_{ij}$ and $\mathbf {r}_{ij_{0}}$ are the position vectors of *j*-th residue referred to the *i*-th one in actual and reference configurations, respectively, while $r_{g_{0}}$ is the same constant already adopted above. Finally ${\varvec{\iota }}$ is a vector with all components equal to 1. The scheme describes only harmonic-like non-periodic interactions (Bacci and Mariano 2021) and overcomes the limits emerging in the approach based on an affine approximation for the velocity of protein residues, the one introduced above. However, this last scheme requires the computation of several quantities at every time step, considerably reducing efficiency of numerical codes. Figure 2 describes how a resulting ellipsoid approximates the free protein unfolding.

Such analyses on a single protein address us in modeling at continuum scale a protein complex.

## Continuum scale description of protein complexes under large strains

### Geometry, motions, and observers

Take two copies of the three-dimensional real space, namely $\mathbb {R}^{3}$ and $\tilde{\mathbb {R}}^{3}$. They are identified by the map $\iota :\mathbb {R}^{3}\longrightarrow \tilde{\mathbb {R}}^{3}$ and are endowed with non-singular metrics *g* and $\tilde{g}$, respectively. A bounded, simply connected region $\mathcal {B}\subset \mathbb {R}^{3}$, with piecewise Lipschitz boundary, represents a macroscopic reference shape for a protein complex we look at. The letter *x* now labels points in $\mathcal {B}$.

Orientation-preserving differentiable one-to-one maps $x\longmapsto y:=\tilde{y}(x)\in \tilde{\mathbb {R}}^{3}$ describe deformations. *F* indicates the derivative $D\tilde{y}(x)$. The gradient of $\tilde{y}$ is given by $\nabla \tilde{y}(x)=D\tilde{y}(x)g^{-1}$ so that $\nabla \tilde{y}(x)$ and $D\tilde{y}(x)$ coincide when $\tilde{g}$ is flat (i.e., it coincides with the identity tensor with both covariant components, so it refers to a orthonormal Cartesian frame of reference). Having in mind the distinction between $\nabla \tilde{y}(x)$ and $D\tilde{y}(x)$, we continue to adopt the standard nomenclature for *F* and call it a *deformation gradient*. As a linear operator, *F* brings with it two its variants: the *formal adjoint* $F^{*}$ and the *transpose* $F^{\mathsf {T}}$ of *F*. Their relation is $F^{\mathsf {T}}=\hat{g}^{-1}F^{*}g$, so that they coincide when both metrics are flat. Also, the requirement that $\tilde{y}$ be orientation-preserving implies, as it is well-known, the constraint $\det F>0$.

A gross motion is a differentiable space-time map $(x,t)\longmapsto y:=\tilde{y}(x,t)\in \tilde{\mathbb {R}}^{3}$, with *t*, the time, ranging in some interval $[0,\bar{t}]$ of the real line. We write here $\dot{y}$ for the velocity in referential description, namely $\dot{y}:=\frac{\mathrm {d}\tilde{y}(x,t)}{\mathrm {d}t}$. It coincides with its Eulerian representation *v*(*y*, *t*). Also, we set $\dot{y}$ to be coincident with $\mathrm {v}:=\tilde{\mathrm {v}}(x,t)$ appearing in the decomposition (1), now considered as a field over $\mathcal {B}$. Consequently, we have

$$\begin{aligned} \dot{y}=\mathrm {v}=v\;. \end{aligned}$$

In other words, the presence of $\mathrm {v}$ in the identities above means that in the continuum modeling of the protein complex we are assigning to each point of $\mathcal {B}$ information pertaining to an entire protein. In this view, an additional space-time differentiable field $(x,t)\longmapsto \nu :=\tilde{\nu }(x,t)\in \mathrm {Hom}(\mathbb {R}^{3},\tilde{\mathbb {R}}^{3})$ is such that its time derivative coincides at every *x* and *t* with $\dot{\nu }(x,t)$ in the decomposition (1).

The pair $(\tilde{y}(x,t), \tilde{\nu }(x,t))$ describes a generalized observable motion.

An *observer* is here a collection of frames assigned to all spaces that are necessary to describe the morphology of a body and its motion (see Mariano 2014 for details on such a general view); so, an observer includes frames on $\mathrm {Hom}(\mathbb {R}^{3},\tilde{\mathbb {R}}^{3})$.

Let $\mathcal {O}$ and $\mathcal {O}'$ be two such observers. Consider the pertinent frames in the ambient space $\mathbb {R}^{3}$. Assume that they are related by time-dependent and time-differentiable isometries; so a place *y*(*x*, *t*) for a material element recorded by $\mathcal {O}$ is seen by $\mathcal {O}'$ as $y'(x,t)=w(t)+y_{0}+Q(t)(y(x,t)-y_{0})$, where $w(t)\in \mathbb {R}^{3}$ and $Q(t)\in SO(3)$. The two observers record two different velocities: $\dot{y}$ for $\mathcal {O}$, $\dot{y}'=\dot{w}+\dot{Q}(y-y_{0})+Q\dot{y}$ for $\mathcal {O}'$. By pulling back $\dot{y}'$ into the frame of the observer $\mathcal {O}$ through $Q^{\mathrm {T}}=Q^{-1}$, we get

$$\begin{aligned} \dot{y}^{\diamond }:=Q^{\mathrm {T}}\dot{y}'=\mathfrak {c}+q\times \left( y-y_{0}\right) +\dot{y}\;, \end{aligned}$$

where $\mathfrak {c}:=Q^{\mathrm {T}}\dot{w}$ is a translation velocity, *q* a rotational one, the characteristic vector of the skew-symmetric tensor $Q^{\mathrm {T}}\dot{Q}$, both depending on time only, and $\times$ indicates the vector product. This analysis is standard.

However, rotating the frames in the physical space $\mathbb {R}^{3}$ alters a perception of the affine motion described by $\nu$ because a rotating observer perceives a rotated protein. Consequently, we need to consider the action of *SO*(3) over $\mathrm {Hom}(\mathbb {R}^{3},\tilde{\mathbb {R}}^{3})$, the manifold on which we read $\nu$, with components $\nu ^{i}_{j}$, both components living in $\tilde{\mathbb {R}}^{3}$, $\mathbb {R}^{3}$ as suggested by the decomposition (1). Then, a counterpart of $\dot{y}^{\diamond }$ is

$$\begin{aligned} \dot{\nu }^{\diamond }=\dot{\nu }+\mathcal {A}\left( \nu \right) q, \end{aligned}$$

where the linear operator $\mathcal {A}\left( \nu \right)$ is a third-rank tensor given by

12 $$\begin{aligned} \mathcal {A}=\nu\bar{\mathbf {e}}-\bar{\mathbf {e}}\nu\;, \end{aligned}$$

where $\bar{\mathbf {e}}$ is Ricci’s alternating index in $\mathbb {R}^{3}$ (see also Mariano 2014 for the proof and further remarks on this treatment of changes in observers).

### Actions, invariance, and balance

The role of observers appears evident already when we discuss the nature of interactions among body elements and with the environment. We call a *part* of $\mathcal {B}$ any regularly open connected subset $\mathfrak {b}$ of $\mathcal {B}$ with piecewise Lipschitz boundary. We subdivide external actions on it into bulk and contact ones. They are defined by the power $\mathcal {P}^{ext}_{\mathfrak {b}}$ that they perform on pairs $(\dot{y}, \dot{\nu })$. We define such a power by

$$\begin{aligned} \mathcal {P}_{\mathfrak {b}}^{ext}\left( \dot{y},\dot{\nu }\right) :=\int _{ \mathfrak {b}}\left( b^{\ddagger }\cdot \dot{y}+\beta ^{\ddagger }\cdot \dot{ \nu }\right) \mathrm {d}\mu (x)+\int _{\partial \mathfrak {b}}\left( \mathfrak {t}_{\partial }\cdot \dot{y} +\tau _{\partial } \cdot \dot{\nu }\right) \mathrm {d}\mathcal {H}^{2}(x), \end{aligned}$$

where $\mathrm {d}\mu (x)$ and $\mathrm {d}\mathcal {H}^{2}(x)$ indicate volume and surface measures, respectively. The subscript $\partial$ indicates that the contact actions $\mathfrak {t}$ and $\tau$ depend on the boundary $\partial \mathfrak {b}$ besides *x* and *t*. Here the bulk action $b^{\ddagger }$ plays the role of $\mathbf {f}^{\ddagger }$ in the previous section. We write $b^{\ddagger }$ instead of $\mathbf {f}^{\ddagger }$ to distinguish the bulk actions as a field over the whole complex from the total force on a single protein, not embedded in a cluster.

We consider balanced those actions for which the external power is invariant under rigid-body-type changes in observers, i.e., those for which

$$\begin{aligned} \mathcal {P}_{\mathfrak {b}}^{ext}\left( \dot{y},\dot{\nu }\right) =\mathcal {P} _{\mathfrak {b}}^{ext}\left( \dot{y}^{\diamond },\dot{\nu }^{\diamond }\right) \end{aligned}$$

for any choice of $\mathfrak {c}$ and *q*, but also for any part $\mathfrak {b}$. The requirement implies the standard balance of forces

$$\begin{aligned} \int _{\mathfrak {b}}b^{\ddag }\;\;\mathrm {d}\mu (x)+\int _{\partial \mathfrak {b}}\mathfrak {t}_{\partial }\;\;\mathrm {d} \mathcal {H}^{2}(x)=0, \end{aligned}$$

and a non-standard balance of couples

$$\begin{aligned} \int _{\mathfrak {b}}\left( \left( y-y_{0}\right) \times b^{\ddag }+\mathcal {A} ^{*}\beta ^{\ddag }\right) \mathrm {d}\mu (x)+\int _{\partial \mathfrak {b}}\left( \left( y-y_{0}\right) \times \mathfrak {t}_{\partial }+\mathcal {A}^{*}\tau _{\partial } \right) \mathrm {d}\mathcal { H}^{2}(x)=0, \end{aligned}$$

where $\mathcal {A}^{*}$ is the formal adjoint of $\mathcal {A}$.

- If $\left| b^{\ddag }\right|$ is bounded over $\mathcal{B}$ and $\mathfrak {t}_{\partial }$ depends continuously on *x*, we can prove that the action-reaction principle holds on flat boundaries. Then, one may further show that, more generally, $\mathfrak {t}_{\partial }$ depends on $\mathfrak {\partial \mathfrak {b}}$ only through the normal *n* to it in all points where *n* is well-defined, i.e., $\mathfrak {t}_{\partial }=\mathfrak {t}:=\tilde{\mathfrak {t}}\left( x,n\right) =-\tilde{\mathfrak {t}}\left( x,-n\right)$. Also, as a function of *n*, $\tilde{\mathfrak {t}}$ is homogeneous and additive, i.e., there exists a second-rank tensor field $x\longmapsto P\left( x\right)$ such that $\tilde{\mathfrak {t}}\left( x,n\right) =P\left( x\right) n\left( x\right)$. This is the standard Cauchy theorem preceded by the Hamel-Noll result; *P* is the *first Piola-Kirchhoff stress*.
- In addition, if $\left| \mathcal {A} ^{*}\beta ^{\ddag }\right|$ is bounded over $\mathcal {B}$ and $\tau _{\partial }$ is continuous with respect to *x*, the microstructural contact action $\tau _{\partial }$ depends on $\mathfrak {\partial \mathfrak {b}}$ only through the normal *n* to it in all points where *n* is well-defined, i.e., $\tau _{\partial }=\tilde{\tau }(x,t,n)=:\tau$, and satisfies a non-standard action-reaction principle, namely,
  $$\begin{aligned} \mathcal {A}^{*}\left( \tilde{\tau } \left( x,t,n\right) +\tilde{\tau } \left( x,t,-n\right) \right) =0. \end{aligned}$$
  Also, as a function of *n*, $\tilde{\tau }$ is homogeneous and additive, i.e., there exists a second-rank tensor field $x\longmapsto \mathcal {S}\left( x\right)$, which we call *microstress*, such that $\tilde{\tau } \left( x,n\right) =\mathcal {S}\left( x\right) n\left( x\right)$. The proof follows a path analogous to the one that is appropriate for the previous item. (Notice that the presumed regularity of $\mathfrak {t}$ and $\tau$ with respect to the space variable can be weakened, obtaining analogous results; in essence, we should need just that the bulk terms are represented by a signed Radon measure and the boundary terms are bounded; in this case the resulting stresses are $L^{\infty }$-maps with respect to the state variables; see, e.g., Dafermos 2005, Ch. 1 for a proof of the standard Cauchy theorem under these weakened assumptions.)
- If both stress fields are in $C^{1}\left( \mathcal {B}\right) \cap C\left( \bar{\mathcal {B}}\right)$ and the bulk actions $x\longmapsto b^{\ddagger }$, $x\longmapsto \beta ^{\ddag }$ are continuous over $\mathcal {B}$, the point-wise balance of forces
  13 $$\begin{aligned} \mathrm {Div}P+b^{\ddag }=0 \end{aligned}$$
  holds and there exists a field $x\longmapsto z\left( x\right) \in T_{\nu }^{*}\mathcal {M}$ such that
  14 $$\begin{aligned} \mathrm {Div}\mathcal {S}+\beta ^{\ddag }-z=0\;,\qquad \mathrm {skw}PF^{*}=\frac{1}{2}\mathsf {e}\left( \mathcal {A}^{*}z+\left( D\mathcal {A}^{*}\right)^{t} \mathcal {S}\right) \;, \end{aligned}$$
  where the superscript ^*t*^ indicates minor right transposition; moreover,
  15 $$\begin{aligned} \mathcal {P}_{\mathfrak {b}}^{ext}\left( \dot{y},\dot{\nu }\right) =\int _{ \mathfrak {b}}\left( P\cdot \dot{F}+z\cdot \dot{\nu }+\mathcal {S}\cdot \dot{N} \right) \mathrm {d}\mu (x)\;. \end{aligned}$$
  We call the right-hand side integral a *internal *(or *inner*) *power*.

In principle, $b^{\ddag }$ and $\beta ^{\ddag }$ include inertial and non-inertial components. Here we accept for $b^{\ddag }$ such a decomposition, namely,

$$\begin{aligned} b^{\ddag }=b^{in}+b\;, \end{aligned}$$

where the superscript *in* labels the inertial component. Unless experimental evidences will dictate the opposite, we presently exclude that relative vibrations among neighboring proteins linked together to form the complex might have peculiar microstructural inertial effects, each one relatively to the rest. As a consequence, in the theoretically admissible additive decomposition $\beta ^{\ddag }=\beta ^{in}+\beta$, which is analogous to the one of $b^{\ddagger }$, we set $\beta ^{in}=0$. Non-inertial bulk actions, directly acting on the microstructure and represented by $\beta$, can be due to the action of radiative fields, even when such actions are obtained by inserting the protein into polarizable water as in Drude 2013 force field (see Kamenik et al. 2020).

Thus, in the present scheme just $b^{in}$ remains as an inertial action. It is defined once again by a relation stating that the time rate of the kinetic energy associated with the gross motion equals the negative of its power on any part of the protein complex and any velocity field, namely

$$\begin{aligned} \frac{\mathrm {d}}{\mathrm {d}t}\int _{\mathfrak {b}}\frac{1}{2}\rho |\dot{y}|^{2}\;\mathrm {d}\mu (x)= -\int _{\mathfrak {b}}b^{in}\cdot \dot{y}\;\mathrm {d}\mu (x)\;. \end{aligned}$$

Of course, the arbitrariness of the velocity implies the standard identification $b^{in}=-\rho \ddot{y}$. Also, since we have identified $\dot{y}$ with the average molecular velocity $\mathrm {v}$ in the decomposition (1), we have also

$$\begin{aligned} b^{in}=-\rho \dot{\mathrm {v}}\;. \end{aligned}$$

### Boundary conditions at continuum scale

Applied forces along the boundary $\partial \mathcal {B}$ are sustained at continuum scale by *Pn* in all points where the normal *n* orienting locally $\partial \mathcal {B}$ is well defined. In terms of the discrete scheme, it is (figuratively) like a single layer ring of molecules, each considered as a material point, would surround $\mathcal {B}$ remaining linked with some points of $\partial \mathcal {B}$. These links simulate the effects of applied forces. In fact, we may imagine a loading device able to prescribe a force $\bar{\mathfrak {t}}=Pn$ along $\partial \mathcal {B}$. On the other side, we find it hard to imagine a device assigning along $\partial \mathcal {B}$ the micro-traction $\tau$, so a natural choice is to prescribe $Sn=0$ along $\partial \mathcal {B}$ in continuum-scale simulations. A boundary condition in terms of *u* appears natural, while prescribing $\nu$ is easy from a mathematical viewpoint but not so immediate in terms of physical feasibility.

## Identification of continuous stresses from the discrete structure

In order to identify *P* and $\mathcal {S}$ as functions of the inter-protein actions, we look at circumstances in which there is a spatial scale at which we can consider the protein complex to be statistically periodic. Then, as a matter of modeling, we select in space a box $\mathbf {e}$ with length side the scale considered. Such a box should contain at least a protein and the links with its neighbor.

Let Greek indices denote different proteins. According to formula (1), the velocity $\mathbf {w}_{\alpha i}$ of the *i*-th amino acid belonging to the $\alpha$-th protein, individuated by the vector $\mathbf {r}_{\alpha i}$, issued by the protein mass center, is given by

16 $$\begin{aligned} \mathbf {w}_{\alpha i}(\mathbf {r}_{\alpha i},x,t)=\mathrm {v}(x,t) + \dot{\nu }(x,t)\mathbf {r}_{\alpha i} + c_{\alpha i}(\mathbf {r}_{\alpha i},x,t)\;, \end{aligned}$$

where $\mathrm {v}$ and $\dot{\nu }$ represent the pertinent fields at continuum scale. The mass center of $\mathbf {e}$ is placed at *x* in the instant *t*. Consider two proteins $\alpha$ and $\beta$, with $\mathbf {r}_{\alpha \beta }$ the vector connecting their centers of mass ($\mathbf {r}_{\alpha \beta }=-\mathbf {r}_{\beta \alpha }$). Take $\bar{\epsilon }$ as smallest diameter of an ellipsoid containing the two proteins. If an amino acid at $\mathbf {r}_{\alpha i}$ is linked with one at $\mathbf {r}_{\beta j}$, we assume that

$$\begin{aligned} |\mathbf {r}_{\alpha i}-\mathbf {r}_{\beta j}|=o(\bar{\epsilon }^{2})\;, \end{aligned}$$

by taking this relation as a definition of complex that can be described at continuum scale, when we strictly refer the condition to first neighbor protein pairs. Consequently, by neglecting addenda where $o(\bar{\epsilon }^{2})$ terms appear, we take

$$\begin{aligned} \mathbf {w}_{\alpha i}-\mathbf {w}_{\beta j}=(D \mathrm {v})\mathbf {r}_{\alpha \beta }+((D \dot{\nu })^{t}\mathbf{r}_{\alpha\beta})\hat{\mathbf{r}}_{\alpha i}+(D c)\hat{\mathbf{r}}_{\alpha i}\;, \end{aligned}$$

where we look here at *c* as a fluctuation field *c*(*x*, *t*) at continuum scale. According to the notation adopted for the product of a second-rank tensor and a vector, here, $((D \dot{\nu })^{t}\mathbf{r}_{\alpha\beta})\hat{\mathbf{r}}_{\alpha i}$ is given in components by $(D \dot{\nu })^{t\,h}_{\;\;Lk}\mathbf{r}^{k}_{\alpha\beta}\hat{\mathbf{r}}_{\alpha i}^{L}=(D \dot{\nu})^{t\,h}_{\;\;Lk}\mathbf{r}^{k}_{\alpha\beta}\mathbf{r}_{\alpha i}^{h}(F^{-\ast})_{h}^{\;L}$, where, once again, we sum over repeated indices (notice that Greek indices and the index *i* in the last formula do not indicate components, while capital indices refer to the reference configuration).

We write $\mathbf {t}_{ij}$ for the (vector) interaction force between the amino acid at $\mathbf {r}_{\alpha i}$ and the one at $\mathbf {r}_{\beta j}$. Write $Cont_{\alpha }$ for the set indexing all proteins linked with the $\alpha -$th one, $Cont(\alpha ,\beta )$ for the set containing pairs (*i*, *j*) of indices denoting amino acids of the $\alpha -$th protein (first index) linked with those of the $\beta -$th one, and $Pr(\mathbf {e})$ for the index set of proteins in $\mathbf {e}$. Also, we indicate by $\mathbf {h}_{(kh)_{\alpha }}$ the interaction force between *h*-th and *k*-th amino acids within the $\alpha$-th protein in $\mathbf {e}$. By $Cont(\alpha k)$ we indicate a set indexing those amino-acids in the $\alpha$-th protein that are connected with the *k*-th amino-acid in the same protein. Then, the *internal power* $\mathcal {P}^{int}$ pertaining to $\mathbf {e}$ is the sum

$$\begin{aligned} \mathcal {P}^{int}:=\mathcal {P}^{self}+\mathcal {P}^{inter}\;, \end{aligned}$$

where $\mathcal {P}^{self}$ is the *self-power* defined by

$$\begin{aligned} \mathcal {P}^{self}:=\sum _{\alpha \in Pr(\mathbf {e})}\sum _{k=1}^{N_{\alpha }}\sum _{h\in Cont(\alpha k)}\mathbf {h}_{(kh)_{\alpha }}\cdot (\dot{\nu }(x,t)\mathbf {r}_{\alpha i} + c_{\alpha i}(\mathbf {r}_{\alpha i},x,t))\;, \end{aligned}$$

where $N_{\alpha }$ is the number of amino acids within the $\alpha$-th protein, while $\mathcal {P}^{inter}$ is the *interaction power* defined by

$$\begin{aligned} \mathcal {P}^{inter}:=\sum _{\alpha \in Pr(\mathbf {e})}\sum _{\beta \in Cont_{\alpha }}\sum _{(i,j)\in Cont(\alpha ,\beta )}\mathbf {t}_{ij}\cdot (\mathbf {w}_{\alpha i}-\mathbf {w}_{\beta j})\;. \end{aligned}$$

By using formula (16), for the fluctuation component $\mathcal {P}^{inter-fluc}$ of $\mathcal {P}^{inter}$ we get

$$\begin{aligned} \mathcal {P}^{inter-fluc}:=\sum _{\alpha \in Pr(\mathbf {e})}\sum _{\beta \in Cont_{\alpha }}\sum _{(i,j)\in Cont(\alpha ,\beta )}\mathbf {t}_{ij}\cdot (D c)\hat{\mathbf{r}}_{\alpha i}\;. \end{aligned}$$

We have also a fluctuation component of the self-power, namely $\mathcal {P}^{self-fluc}$, given by

$$\begin{aligned} \mathcal {P}^{self-fluc}:=\sum _{\alpha \in Pr(\mathbf {e})}\sum _{k_{\alpha }=1}^{N_{\alpha }}\sum _{h\in Cont(\alpha k)}\mathbf {h}_{(kh)_{\alpha }}\cdot c_{\alpha i}(\mathbf {r}_{\alpha i},x,t))\;. \end{aligned}$$

To identify the stresses *P* and $\mathcal {S}$, as discrete-to-continuum equivalence criterion, we adopt an appropriate version of (7), namely we impose

17 $$\begin{aligned} |\mathbf {e}|^{-1}(\mathcal {P}^{int}-\mathcal {P}^{self-fluc}-\mathcal {P}^{int-fluc}) =P\cdot D \mathrm {v}+z\cdot \dot{\nu }+\mathcal {S}\cdot D\dot{\nu }, \end{aligned}$$

presuming that it holds for any choice of $D\mathrm {v}$, $\dot{\nu }$, and $D\dot{\nu }$. Such an arbitrariness implies the explicit expressions of stresses and self-actions in terms of geometry and interaction forces at molecular level:

18 $$\begin{aligned} P&=\frac{1}{|\mathbf {e}|}\sum _{\alpha \in Pr(\mathbf {e})}\sum _{\beta \in Cont_{\alpha }}\sum _{(i,j)\in Cont(\alpha ,\beta )}\mathbf {t}_{ij}\otimes \hat{\mathbf{r}}_{\beta \alpha }\;, \end{aligned}$$

19 $$\begin{aligned} z&=\frac{1}{|\mathbf {e}|}\sum _{\alpha \in Pr(\mathbf {e})}\sum _{k_{\alpha }=1}^{N_{\alpha }}\sum _{h\in Cont(\alpha k)}\mathbf {h}_{(kh)_{\alpha }}\otimes \mathbf {r}_{\alpha h}\;, \end{aligned}$$

20 $$\begin{aligned} \mathcal {S}&=\frac{1}{|\mathbf {e}|}\sum _{\alpha \in Pr(\mathbf {e})}\sum _{\beta \in Cont_{\alpha }}\sum _{(i,j)\in Cont(\alpha ,\beta )}\mathbf {t}_{ij}\otimes \mathbf {r}_{\alpha i}\otimes \hat{\mathbf{r}}_{\beta \alpha }\;. \end{aligned}$$

These expressions, written here in terms of fields defined over the reference place, have their Eulerian counterpart given by

21 $$\begin{aligned} \sigma&=\frac{1}{|\mathbf {e}|\det F}\sum _{\alpha \in Pr(\mathbf {e})}\sum _{\beta \in Cont_{\alpha }}\sum _{(i,j)\in Cont(\alpha ,\beta )}\mathbf {t}_{ij}\otimes \mathbf {r}_{\beta \alpha }\;, \end{aligned}$$

22 $$\begin{aligned} z_{a}&=\frac{1}{|\mathbf {e}|\det F}\sum _{\alpha \in Pr(\mathbf {e})}\sum _{k_{\alpha }=1}^{N_{\alpha }}\sum _{h\in Cont(\alpha k)}\mathbf {h}_{(kh)_{\alpha }}\otimes \mathbf {r}_{\alpha h}\;, \end{aligned}$$

23 $$\begin{aligned} \mathcal {S}_{a}&=\frac{1}{|\mathbf {e}|\det F}\sum _{\alpha \in Pr(\mathbf {e})}\sum _{\beta \in Cont_{\alpha }}\sum _{(i,j)\in Cont(\alpha ,\beta )}\mathbf {t}_{ij}\otimes \mathbf {r}_{\alpha i}\otimes \mathbf{r}_{\beta \alpha }\;. \end{aligned}$$

In selecting the equivalence criterion (17), we relegate to heat the effects of fluctuations. As an alternative and restricted choice, we could reduce the description of protein complex mechanics just to what pertains the average velocity $\mathrm {v}$ of each protein. In this way the possible independent homogeneous strain around the protein mass center, measured by $\nu$, would fall within fluctuations and we would not have both *z* and $\mathcal {S}$, reducing ourselves to a standard continuum format (Cauchy-type) rather than adopting a multi-field scheme as we have done here. The choice between such two possible paths is a matter of modeling. It depends on how much details we are interested to consider in describing the mechanics of protein complexes.

In this setting the vector *b*, which represents non-inertial body forces, has to be considered as the sum of two components: the standard gravitational action and the cumulative effect of other environmental actions on each amino-acid, such as the driving force exerted by a fluid over amino-acids. This last contribution is given by the first addendum in the right-hand side part of formula (8), namely $|\hat{\mathbf {e}}|^{-1}\sum _{i=1}^{N} \mathbf {f}_{i}$.

## Constitutive restrictions at continuum scale

The second law of thermodynamics limits a priori constitutive choices. Here we restrict our analysis to the isothermal setting (a condition obtained also in molecular simulations) and write the second law in terms of a mechanical dissipation inequality, which reads

$$\begin{aligned} \frac{\mathrm {d}}{\mathrm {d}t}\int _{\mathfrak {b}}\psi \;\;\mathrm {d}\mu (x)-\int _{ \mathfrak {b}}\left( P\cdot \dot{F}+z\cdot \dot{\nu }+\mathcal {S}\cdot \dot{N} \right) \mathrm {d}\mu (x)\le 0\;, \end{aligned}$$

where $\psi$ is the free energy density. We presume it holds true for any choice of the time rates involved. Then, we select the following constitutive functional dependence on the state variables:

$$\begin{aligned} \psi&=\tilde{\psi }(F,\nu , N)\;,\quad P=\tilde{P}(F,\nu , N)\;,\quad \mathcal {S}=\tilde{\mathcal {S}}(F,\nu , N)\;, \\ z&=z^{e}+z^{d}:=\tilde{z}^{e}(F,\nu , N)+\tilde{z}^{d}(F,\nu , N; \dot{F}, \dot{\nu }, \dot{N})\;, \end{aligned}$$

where *e* and *d* in superscript position indicate, respectively, energetic and dissipative components of the self-action *z*. Precisely, we consider dissipative effects but restrict them only to the self-action that every protein in the complex exerts over itself. Of course, generalizations are possible: they may include macroscopic bulk viscosity, which implies an additive decomposition of *P* into energetic and dissipative components, as for *z*, or just viscosity at the microstress ($\mathcal {S})$ level, with analogous decomposition, or both. We do not explore in detail here such possibilities.

By inserting into the inequality and exploiting the arbitrariness of $\dot{F}$, $\dot{\nu }$, and $\dot{N}$, we get

$$\begin{aligned} P=\frac{\partial \psi }{\partial F}\;,\quad z^{e}=\frac{\partial \psi }{\partial \nu }\;, \quad \mathcal {S}=\frac{\partial \psi }{\partial N}\;,\quad z^{d}\cdot \dot{\nu }\ge 0\;. \end{aligned}$$

The last inequality is compatible with

$$\begin{aligned} z=\frac{\partial \psi }{\partial \nu }+a_{\nu }(\dots )\dot{\nu }\;, \end{aligned}$$

where $a_{\nu }(\dots )$ is a positive-valued state function, which can reduce to a scalar, as in the Introduction.

The stresses *P*, $\mathcal {S}$, and the self-action *z* are fields defined over the reference configuration $\mathcal {B}$. Since the deformation map $\tilde{y}$ is one-to-one, they have their counterparts as fields defined in the current configuration $\mathcal {B}_{c}:=\tilde{y}(\mathcal {B},t)$, namely

$$\begin{aligned} \sigma =\frac{1}{\mathrm {det} F}\frac{\partial \psi }{\partial F}F^{*}\;,\quad z_{c}=\frac{1}{\mathrm {det} F}\big (\frac{\partial \psi }{\partial \nu }+a_{\nu }(\dots )\dot{\nu }\big )\;,\quad \mathcal {S}_{c}=\frac{1}{\mathrm {det} F}\frac{\partial \psi }{\partial N}F^{*}, \end{aligned}$$

where $\sigma$ is the standard *Cauchy stress*.

### Remark 1

Here, the second law has been used in a traditional way (the one indicated in reference Coleman and Noll 1959), i.e., as a source of constitutive restrictions. However, the whole continuum structure proposed in Sect. 3 and the present one could be fully derived from the second law (including balances and the representation of contact actions, i.e., Cauchy’s theorem) once we consider changes in observers ruled by diffeomorphisms not necessarily coinciding with isometries (or induced by isometries as it occurs in the formula (12)) and supplement the second law with the so-called *covariance principle*, which prescribes that if for any given observer a process is dissipative, any other observer must perceive that process as a dissipative one. We do not into details here, leaving the discussion to another work, which would be based on an appropriate generalization of the path followed to obtain the analogous result in finite-strain plasticity in reference (Mariano 2013).

## Symmetry and lack of symmetry for the Cauchy stress

Write $u:=\tilde{u}(x,t)=\tilde{y}(x,t)-\iota (x)$ for the *displacement field*, so that $F=D u+I$, with the second-rank unit tensor. The condition

$$\begin{aligned} |D u|<<1 \end{aligned}$$

defines what we commonly call a *small strain* regime. When it occurs, we may avoid to distinguish between referential ($\mathcal {B}$) and current ($\mathcal {B}_{c}:=\tilde{y}(\mathcal {B},t)$) configurations, so that

$$\begin{aligned} \sigma \approx P\;,\quad z_{c}\approx z\;,\quad \mathcal {S}_{c}\approx \mathcal {S}. \end{aligned}$$

Under these conditions we can admit for the free energy a quadratic form

24 $$\begin{aligned} \psi (D u, \nu , D\nu )=\mathrm {Quad}^{+}(D u, \nu , D\nu )\;, \end{aligned}$$

where $\mathrm {Quad}^{+}$ indicates generically a quadratic form with positive-definite coefficients.

Such a specific energy form, holding in small strain regime, and the constitutive restrictions deduced previously imply

$$\begin{aligned} |z|\propto |\nu |+|Du|\;,\qquad |\mathcal {S}|\propto |D\nu | \end{aligned}$$

at least when the protein complex portion in the box $\mathbf {e}$ is center-symmetric so that odd tensor coefficient in the quadratic form vanish (for appropriate remarks on center-symmetry see, e.g., reference Nemat-Nasser and Hori 1998). Also, from the expression (12) we also deduce that $|\mathcal {A}|$ behaves as $|\nu |$, the local balance of couples (14)$_{2}$ implies the following proposition:

### Proposition 1

The protein self-action *z* and the microstress $\mathcal {S}$ break Cauchy’s stress classical symmetry as second-order perturbations, so that in the linear constitutive behavior the stress $\sigma$ remains symmetric.

We interpret such a result by saying that the protein deformation described by $\nu$ (which is *relative* to the neighboring proteins, a fluctuation with respect to the gross strain $F^{\mathsf {T}}F$) induces local couples described by (say) $\xi$, which is the characteristic vector of the skew-symmetric tensor with *ij*-th component given (in an orthogonal frame, where formal adjoint and transpose, indicated as usual by the apex $\mathsf{T}$, coincide) by

$$\begin{aligned} \bar{\mathbf {e}}_{ijk}(\mathcal {A}_{knm}z_{mn}+(\nabla _{k}\mathcal {A}_{rnm})\mathcal{S}_{mnr})\;. \end{aligned}$$

In compact form, vector $\xi$ is defined by the relation

$$\begin{aligned} \bar{\mathbf {e}}(\mathcal {A}^{\mathsf {T}}z+(\nabla \mathcal {A}^{\mathsf{T}})^{t}\mathcal{S})a=\xi \times a\;, \end{aligned}$$

for any vector *a*.

## Equilibrium configurations under large strains—native states

We commonly think that native states of single proteins are thermodynamically stable at physiological conditions. So, they are identified by most favorable minima in a free energy landscape. A question is how such a general conviction has to be interpreted within the scheme we present here. In it, the determination of native states then reduces to the analysis of a minimum problem:

25 $$\begin{aligned} \begin{aligned} minimize&\bigg (\int _{\mathcal {B}}\psi (F,\nu , N)\;\mathrm {d}\mu(x)=\int _{\mathcal {B}}\psi (D\tilde{y}(x),\tilde{\nu }(x), D\tilde{\nu }(x))\;\mathrm {d}\mu(x)\bigg )\\&\text {for}\quad \tilde{y}\in X\quad \text {and}\quad \tilde{\nu }\in Y\;, \end{aligned} \end{aligned}$$

under, e.g., Dirichlet’s boundary conditions, i.e., under prescribed $\tilde{y}$ and $\tilde{\nu }$ over the boundary, or portions of it, as specified below.

To tackle the problem, we need to specify, as constitutive choices, the two functional spaces *X*, *Y*, and the structure of $\psi$ as a function.

For this last aspect, first we point out that when large strains occur, selecting the free energy to be a convex function of the deformation gradient would be incompatible with the physical assumption that the free energy is objective, i.e., invariant under rigid-body-type changes in observers, as pointed out by B. Coleman and W. Noll in 1959 (Coleman and Noll 1959). The closest constitutive choice to convexity, which avoids the physical incompatibility just mentioned, is to presume that the free energy is polyconvex with respect to *F*, i.e., it is a convex function of the triple $(F,\mathrm {cof}F, \det F)$, where here $\mathrm {cof}F:=(\det F)F^{-\mathsf {T}}$ is the cofactor of *F*; it measures changes in oriented areas. The same incompatibility does not occurs for the dependence of the free energy on *N*, which we can select as a convex function of *N*, as we do here.

We follow a path indicated in reference Mariano and Modica (2009) in the more general setting in which $\nu$ belongs to a finite-dimensional differentiable manifold not necessarily coinciding with a linear space.

### Functional choices for the spaces *X* and *Y*

For *X* we select the space of weak diffeomorphisms, a function class introduced in reference Giaquinta et al. (1989). Its definition requires the introduction of preliminary notions.

First recall that for any two vectors *a* and *b*, the wedge product $a\wedge b$ is the skew-symmetric part of the dyad $a\otimes b$. The definition can be naturally extended to higher-order skew products; for example $a\wedge b\wedge c$ is the fully skew-symmetric component of $a\otimes b\otimes c$; so $a\wedge b\wedge c$ belongs to the space of fully contravariant (indexes in upper position) third-rank skew-symmetric tensors, a space that we indicate by ${\Lambda} _{3}({\mathbb {R}}^{3}\times {\tilde{\mathbb {R}}}^{3})$. A generic element *M* is determined by two constants, say $\zeta$, $\mathfrak {a}$, and two second-rank linear operators, say *H*, *A*, which can be in principle independent. With respect to bases $\left( e_{1},e_{2},e_{3}\right)$ and $\left( \tilde{e}_{1},\tilde{e}_{2}, \tilde{e}_{3}\right)$ in $\mathbb {R}^{3}$ and $\tilde{\mathbb {R}}^{3}$, respectively, we have

$$\begin{aligned} \begin{aligned} M =\,&\zeta e_{1}\wedge e_{2}\wedge e_{3}+\sum _{i,J}^{3}\left( -1\right) ^{J-1}H^{iJ}e_{\bar{J} }\wedge \tilde{e}_{i} \\&+\sum _{i,J}^{3}\left( -1\right) ^{i-1}A^{iJ}e_{J}\wedge \tilde{e}_{\bar{\imath }}+\mathfrak {a}\tilde{e}_{1}\wedge \tilde{e}_{2}\wedge \tilde{e}_{3}\;, \end{aligned} \end{aligned}$$

where $\bar{J}$ is the complementary multi-index to *J* with respect to $\left( 1,2,3\right)$ and $\bar{\imath }$ has an analogous relation with *i* (for example, if $J=1$, then $\bar{J}=\left( 2,3\right)$ and $e_{\bar{J}}=e_{2}\wedge e_{3}$, and the same holds for the index *i* and its pertinent $\bar{\imath }$). For the sake of conciseness we shall write $M=\left( \zeta ,H,A,\mathfrak {a} \right)$. In particular, we can associate *M* to a single linear operator. Precisely, by choosing, say, *F* for such a linear operator, we have $\zeta =1,\ H=Fg^{-1},$ $A= \tilde{g}^{-1}\mathrm {cof}F$ when $\mathrm {cof}F$ is defined by $\left( \det F\right) \left( F^{-1}\right) ^{*}$ or $A=\text {cof}Fg^{-1}$ when we consider $\mathrm {cof}F$ as given by $\left( \det F\right) \left( F^{-1}\right) ^{\mathsf {T}}$, and $\mathfrak {a}=\det F$. In short we shall write $M\left( F\right) =\left( F,\mathrm {cof}F,\det F\right)$ when $M=M\left( F\right)$ (see the treatise Giaquinta et al. 1998 for further geometrical details and pertinent proofs). When we have kinematic compatibility, i.e., when *F* is precisely the gradient of deformation, i.e., it coincides with $D\tilde{y}(x,t)$, so that $M\left( F\right) =M(Dy)$.

The dual space of ${\Lambda}_{3}(\mathbb {R}^{3}{\times} \tilde{\mathbb{R}}^{3})$, indicated by $\Lambda ^{3}(\mathbb {R}^{3}\times \tilde{\mathbb{R}}^{3})$, is the one of fully covariant third-rank skew-symmetric tensors. A map from $\mathbb {R}^{3}\times \tilde{\mathbb{R}}^{3}$ to $\Lambda ^{3}(\mathbb {R}^{3}\times \tilde{\mathbb{R}}^{3})$ is what we call here a 3-*form*. We’ll indicate by $\mathcal {D}^{3}(\mathbb {R}^{3}\times \tilde{\mathbb{R}}^{3})$ the pertinent space. For $\omega$ one such a form, the duality product by *M* is once again indicated by an interposed dot.

For $u: \mathbb {R}^{3}\longrightarrow \tilde{\mathbb {R}}^{3}$ a smooth map, we define the *current* of *u* as the functional $G_{u}$, defined over $\mathcal {D}^{3}(\mathbb {R}^{3}\times \tilde{\mathbb{R}}^{3})$, with values given by the formula

$$\begin{aligned} G_{u}\left( \omega \right) =\int _{\mathcal {B}}\omega \left( x,u\left( x\right) \right) \cdot M\left( Du\left( x\right) \right) \;\mathrm {d}x \end{aligned}$$

for every smooth $3-$form with compact support in $\mathbb {R}^{3}\times \tilde{\mathbb{R}}^{3}$.

The formula makes sense also when *u* is a $W^{1,1}$ map, provided appropriate specifications. First, recall that measurable functions *f* into topological spaces with a countable basis can be approximated by continuous functions on arbitrarily large portions of their domain (this statement is in essence Lusin’s theorem). If $f:\Omega \rightarrow \tilde{\mathbb {R}}^{3}$ is locally summable in Lebesgue’s sense, by the Lebesgue differentiation theorem almost every *x* in $\Omega$ is a Lebesgue point of *f*, i.e., a point such that for some $\lambda \in \tilde{\mathbb {R}}^{3}$

$$\begin{aligned} \lim _{r\rightarrow 0^+}\frac{1}{|B(x,r)|}\int _{B(x,r)}|f(z)-\lambda |\;\mathrm {d}x=0\;, \end{aligned}$$

with *B*(*x*, *r*) a ball of radius *r*, centered at *x*, which Lebesgue measure is |*B*(*x*, *r*)|. The number $\lambda =f(x)$ is called Lebesgue value of *f* at *x*.

Consider $u\in W^{1,1}(\mathcal {B}, \tilde{\mathbb {R}}^{3})$, and indicate by $\tilde{\mathcal {B}}$ the set of Lebesgue points for both *u* and *Du*. Also, let $\tilde{u}$ be a Lusin representative of *u*. Write $\tilde{u}(x)$ and $D\tilde{u}(x)$ for the Lebesgue values of *u* and *Du* at $x\in \tilde{\mathcal {B}}$. The graph of *u* is now the set

$$\begin{aligned} \mathcal {G}_{u}:=\{(x,y)\in \mathcal {B}\times \tilde{\mathbb {R}}^{3}\;|\;x\in \tilde{\mathcal {B}},\,y:=\tilde{u}(x)\}. \end{aligned}$$

A Lusin-type theorem for $W^{1,1}$ functions assures that $\mathcal {G}_{u}$ is a 3-rectifiable subset of $\mathcal {B}\times \tilde{\mathbb {R}}^{3}$, with approximate tangent space at *x*, *u*(*x*) generated by the vectors $(\mathbf {e}_{1}, Du(x)\mathbf {e}_{1}), (\mathbf {e}_{2}, Du(x)\mathbf {e}_{2}), (\mathbf {e}_{3},Du(x)\mathbf {e}_{3})$. For any $u\in W^{1,1}(\mathcal {B}\times \tilde{\mathbb {R}}^{3})$, with $|M (Du (x))|\in L^{1}(\mathcal {B})$, the *current* of *u*, more specifically a 3-current integration over the graph of *u*, is once again the linear functional $G_{u}$ on smooth 3-forms with compact support in $\mathcal {B}\times \tilde{\mathbb {R}}^{3}$, defined above.

We associate with $G_{u}$ a notion of *boundary current*, indicated by $\partial G_{u}$ and defined by

$$\begin{aligned} \partial G_{u}\left( \omega \right) :=G_{u}\left( d\omega \right) ,\text { \ \ \ }\omega \in \mathcal {D}^{2}\left( \mathcal {B}\times \tilde{\mathbb {R}}^{3}\right) \;, \end{aligned}$$

where *d* indicates differential of forms (if $\omega$ is a *n*-form, $d\omega$ is a $(n+1)$-form). When *u* is smooth, $\partial G_{u}\left( \omega \right) =0$ for all $\omega \in \mathcal {D}^{2}\left( \mathcal {B}\times \tilde{\mathbb {R}}^{3}\right)$. However, for functions $u\in W^{1,1}\left( \mathcal {B},\tilde{\mathbb {R}}^{3}\right)$ with $\left| M\left( Du\left( x\right) \right) \right| \in L^{1}\left( \mathcal {B}_{0}\right)$, the boundary current $\partial G_{u}$ does not vanish in general. Imposing the condition $\partial G_{u}\left( \omega \right) =0$ assures that the graph of $u\in W^{1,1}\left( \mathcal {B},\tilde{\mathbb {R}}^{3}\right)$ has no discontinuities, i.e., vertical components (see for the proof the treatise Giaquinta et al. 1998).

#### Definition 1

A map $u\in W^{1,1}(\mathcal {B}, \tilde{\mathbb {R}}^{3})$ is a *weak diffeomorphism* (formally, $u\in \mathrm {dif}(\mathcal {B}, \tilde{\mathbb {R}}^{3})$) if

1. $$\left| M\left( Du\left( x\right) \right) \right| \in L^{1}(\mathcal {B})$$,
2. $$\partial G_{u}=0$$,
3. $$\det Du(x)>0$$, a.e. $$x\in \mathcal {B}$$,
4. for any $$\mathsf {f}\in C^{\infty }_{c}(\mathcal {B}\times \tilde{\mathbb {R}}^{3}))$$
  $$\begin{aligned} \int _{\mathcal {B}}\mathsf {f}(x,u(x))\det Du(x)\; \mathrm {d}\mu (x)\le \int _{\tilde{\mathbb {R}}^{3}}\sup _{x\in \mathcal {B}}\mathsf {f}(x,u(x))\;\mathrm {d}\mu (x). \end{aligned}$$

The last condition ensures that the $W^{1,1}$-map considered allows frictionless contact among parts of the body boundary while still prevents the penetration of matter (see also remarks and proofs in references Giaquinta et al. (1989) and Giaquinta et al. (1998)). The space of weak diffeomorphisms is our choice for the space *X*. It excludes consideration of possible protein disconnections or relative slips determining irrecoverable strain. Accounting for them would imply to choose weak diffeomorphisms modeled over the space of special maps of bounded variation. We do not pursue here such a choice, which is also possible, indeed, because we are interested here only in the elastic behavior of protein complexes.

The space defined above has closure and compactness.

#### Theorem 2

(Giaquinta et al. 1989)

1. (Closure) Let $\left\{ u_{k}\right\}$ be a sequence in $dif^{1,1}(\mathcal {B},\tilde{\mathbb {R}}^{3})$. If
  $$\begin{aligned} u_{k}\rightharpoonup u\text { \ \ \ and \ \ \ }M\left( Du_{k}\right) \rightharpoonup v \end{aligned}$$
  weakly in $L^{1}$, then $v=M\left( Du\right)$ a.e. and $u\in dif^{1,1}( \mathcal {B},\tilde{\mathbb {R}}^{3})$.
2. (Compactness) Let $\left\{ u_{k}\right\}$ be a sequence with $u_{k}\in W^{1,r}(\mathcal {B},\tilde{\mathbb {R}}^{3})$, $r>1$, and for any *k*, the map $u_{k}$ is also a weak diffeomorphism. Assume that there exists a constant $C>0$ and a convex function $\vartheta :\left[ 0,+\infty \right) \rightarrow \mathbb {R} ^{+}$ such that $\vartheta \left( t\right) \rightarrow +\infty$ as $t\rightarrow 0^{+}$, and
  $$\begin{aligned} \left\| M\left( Du_{k}\right) \right\| _{L^{r}\left( \mathcal {B}\right) }\le C,\text { \ \ \ \ }\int _{\mathcal {B}}\vartheta \left( \mathop \mathrm{det}\nolimits Du_{k}\left( x\right) \right) \text { }\mathrm {d}x\le C. \end{aligned}$$
  Then, by taking subsequences $\left\{ u_{j}\right\}$ with $u_{j}\rightharpoonup u$ in $W^{1,r}(\mathcal {B},\tilde{\mathbb {R}}^{3})$, we have $u_{j}\rightarrow u$ in $L^{r}\left( \mathcal {B}\right)$, $M\left( Du_{j}\right) \rightharpoonup M\left( Du\right)$ in $L^{r}$ and $\int _{\mathcal {B}}\vartheta \left( \mathop \mathrm{det}\nolimits Du\left( x\right) \right)$ $dx\le C$. In particular, *u* is a weak diffeomorphism.

Then, for *X*, the space of deformations that we consider admissible here, we select

$$\begin{aligned} dif^{r,1}(\mathcal {B},\tilde{\mathbb {R}}^{3}):=\left\{ u\in dif^{1,1}( \mathcal {B},\tilde{\mathbb {R}}^{3})|\left| M\left( Du\right) \right| \in L^{r}\left( \mathcal {B}\right) \right\} , \end{aligned}$$

for some $$r>1$$.

Less preliminaries demanding is the functional choice for *Y*, the space of morphological descriptor maps, which we identify with the Sobolev space

$$\begin{aligned} W^{1,s}\big (\mathcal {B}, \mathrm {Hom}(\tilde{\mathbb {R}}^{3},\tilde{\mathbb {R}}^{3})\big ) \end{aligned}$$

for $$s>1$$.

### An existence result

We consider Dirichlet-type boundary conditions. We select open portions $\partial \mathcal {B}_{y}$ and $\partial \mathcal {B}_{\nu }$ of $\partial \mathcal {B}$, which do not necessarily coincide or complement. On them we presume that $\tilde{y}$ and $\tilde{\nu }$ are prescribed in the sense of traces.

Define the space $\mathcal {W}_{r,s}$ as

$$\begin{aligned} \mathcal {W}_{r,s}:=\left\{ \left( \tilde{y},\tilde{\nu } \right) |\tilde{y}\in dif^{r,1}(\mathcal {B},\tilde{\mathbb {R}}^{3}),\;\tilde{\nu } \in W^{1,s}\big (\mathcal {B}, \mathrm {Hom}(\mathbb {R}^{3},\tilde{\mathbb {R}}^{3})\big ) \right\} . \end{aligned}$$

Extend the energy functional in the problem (25) to $\mathcal {W}_{r,s}$ as

$$\begin{aligned} \mathcal {E}( \tilde{y},\tilde{\nu } ) =\int _{\mathcal {B}}\psi \big ( x, \tilde{y}(x), D\tilde{y}(x), \tilde{\nu }(x), D\tilde{\nu }(x)\big )\;\mathrm {d}x, \end{aligned}$$

where $\tilde{y}(x), D\tilde{y}(x), \tilde{\nu }(x), D\tilde{\nu }(x)$ are the Lebesgue values of $\tilde{y}$, $\tilde{\nu }$ and their weak derivatives.

Consider the energy density $\psi$ as a map

$$\begin{aligned} \psi :\mathcal {B}\times \tilde{\mathbb {R}}^{3}\times M_{3\times 3}\times M_{3\times 3}^{+}\times M_{9\times 3}\rightarrow \bar{\mathbb{R}}^{+} \end{aligned}$$

with values $\psi \left( x, y, F,\nu , N\right)$, where $M_{n\times m}$ is the space of $n\times m$ matrices and the superscript $+$ indicates those with positive determinant (defined when $n=m$).

The assumptions below apply.

1. $\psi$ is polyconvex in *F* and convex in *N*. More precisely, there exists a Borel function
  $$\begin{aligned} P\psi :\mathcal {B}\times \tilde{\mathbb {R}}^{3}\times M_{3\times 3}\times \Lambda _{3}(\mathbb {R}^{3}\times \tilde{\mathbb {R}})\times M_{9\times 3}\rightarrow \bar{\mathbb{R}}^{+}, \end{aligned}$$
  with values $P\psi \left( x, y, \nu , \xi , N\right)$, where $N:=D\tilde{\nu }(x)$, which is
  - lower semi-continuous in $\left( y,\nu ,\xi ,N\right)$ for almost every $x\in \mathcal {B}$,
  - convex in $\left( \xi ,N\right)$ for any $\left( x,y,\nu \right)$,
  - such that $P\psi \left( x,y,\nu ,M\left( F\right) , N\right) =\psi \left( x, y,\nu , F, N\right)$ for any list of entries $\left( x, y,\nu , F, N\right)$ with $\mathop \mathrm{det}\nolimits F>0$.
  In terms of $P\psi$, the energy functional becomes
  26 $$\begin{aligned} \mathcal {E}( \tilde{y},\tilde{\nu } ) =\int _{\mathcal {B}}P\psi \big ( x, \tilde{y}(x), M(F), \tilde{\nu }(x), N\big )\;\mathrm {d}x. \end{aligned}$$
2. The energy density $\psi$ satisfies the growth condition
  27 $$\begin{aligned} \psi \left( x,y,\nu ,F\mathbf {,}N\right) \ge C_{1}\left( \left| M\left( F\right) \right| ^{r}+\left| N\right| ^{s}\right) +\vartheta \left( \mathop \mathrm{det}\nolimits F\right) \end{aligned}$$
  for any $\left( x,y,\nu ,F\mathbf {,}N\right)$ with $\mathop \mathrm{det} \nolimits F>0$, $r,s>1$, $C_{1}>0$ constants and $\vartheta :\left( 0,+\infty \right) \rightarrow \mathbb {R}^{+}$ a convex function such that $\vartheta \left( t\right) \rightarrow +\infty$ as $t\rightarrow 0^{+}$.

The stability condition imposed by the convexity of $P\psi$ with respect to the pair $\left( \xi ,N\right)$ implies an interplay between macroscopic strain and microstructural changes of protein shapes within the complex under analysis.

#### Theorem 3

Under the hypotheses (H1) and (H2), if there is a pair $\left( \bar{y},\bar{\nu }\right) \in \mathcal {W}_{r,s}$ such that $\mathcal {E}\left( \tilde{y}_{0},\tilde{\nu } _{0}\right) <+\infty$, the functional $\mathcal {E}$ achieves its minimum in the classes

$$\begin{aligned} \mathcal {W}_{r,s}^{d}:=\left\{ \left( \tilde{y},\tilde{\nu } \right) \in \mathcal {W} _{r,s}|\tilde{y}=\tilde{y}_{0}\text { on }\partial \mathcal {B}_{y},\tilde{\nu } =\tilde{\nu } _{0}\text { on } \partial \mathcal {B}_{\nu }\right\} \end{aligned}$$

and

$$\begin{aligned} \mathcal {W}_{r,s}^{c}:=\left\{ \left( u,\nu \right) \in \mathcal {W}_{r,s} \text { }|\text { }\partial G_{\tilde{y}}=\partial G_{\tilde{y}_{0}}\text { on }\mathcal {D} ^{2}( \mathbb {R}^{3}\times \hat{\mathbb{R}}^{3}),\tilde{\nu } =\tilde{\nu } _{0}\text { on } \partial \mathcal {B}_{\nu }\right\} \,, \end{aligned}$$

where the Dirichlet-type boundary conditions have to be intended in the sense of traces.

#### Proof

The statement follows from the closure and compactness theorem in Sect. (7.1) and De Giorgi’s and Ioffe’s classical semi-continuity results.

Indeed, Theorem 3 is a special case of Mariano-Modica’s existence result for the energy minimization in the general model-building framework for the mechanics of complex materials (Mariano and Modica 2009) (results collected in that paper, where $\nu$ belongs to a differentiable manifold embedded in a linear space, can extend the present result; also we can weaken assumptions on the energy by exploiting the specific circumstance that in the present case $\nu$ belongs to a linear space, by exploiting the path followed by P. Neff in reference Neff 2006; further generalization towards the determination of native states for interacting protein complexes of different species can be obtained by resorting to the general results in reference Focardi et al. 2015).

## Concluding remarks

- The local affine approximation (1) implies a multi-field continuum model that can be interpreted as the one of a micromorphic continuum (so named after proposals in Mindlin (1964), Green and Rivlin (1964), Eringen (1999), not related to proteins but falling as special cases within the general model-building framework for the mechanics of complex materials, the one discussed in references Capriz (1989), Mariano (2002), Mariano (2014)). Here, we find reasons based on direct simulations (those collected in reference Bacci and Mariano (2021)) to consider such a continuum scheme appropriate for describing the mechanics of protein complexes and we are able to identify the actions at continuum scale in terms of those occurring in the protein discrete structure.
- The identification procedure is in terms of power equivalence. It underlines implicitly a general issue: *when we want to homogenize a discrete structure, not always the resulting continuum scheme falls within the traditional format of continuum mechanics.* In other words, a single level periodic lattice can be put into correspondence with the traditional continuum format in which only a region in space describes the body morphology and we go from a configuration to another only by point-valued one-to-one differentiable maps. Multi-lattices or even just the quasi-periodic ones imply multi-field continuum schemes, as the one described here. In addition to placements of lattice-cell-mass-centers, further variables represent peculiarities in the discrete structure or they refer to the pertinent kinematics, as $\nu$ here, where it describes the independent protein homogeneous strain around its center of mass.

In summary, our continuum approach avoids the need of a direct atomistic simulation of the whole complex but strictly retains information of the protein discrete structure.

Continuum scale constitutive relations for $\sigma$ or *P*, $\mathcal {S}_{c}$ or $\mathcal {S}$, and $z_{c}$ or *z* emerge once we prescribe the structure of $\mathbf {t}_{ij}$ and $\mathbf {h}_{(hk)_{\alpha }}$ in terms of $\nu$, $D\nu$, and *F* or *Du*. The identification process above described addresses also us to prescribe at continuum scale boundary conditions, which could be hard to identify otherwise, when referred to $\nu$ and $\mathcal {S}n$, with *n* a normal to the boundary $\partial \mathcal {B}$. A procedure goes as follows:

- First we assign constitutive structures to $\mathbf {t}$ and $\mathbf {h}$ appearing in the formulas for *P*, *z*, $\mathcal {S}$. They depend on how we consider the bonds inside a protein and among neighboring molecules.
- Previous formulas refer to the geometry of molecular arrangements in the cell $\mathbf {e}$, when the complex is statistically periodic at a certain scale.
- Eventually, we have constitutive equations to be used in implementing the balances (13) and (14) through their weak form (15) into finite-element schemes or other computational structures.
- When we have no evidence of a statistical homogeneity beyond a certain (small but greater than molecular size) spatial scale, we choose a geometry and consider stochastic both the number of contacts and (as a possible further step) the constitutive properties of bonds. A set of several simulations with different geometries becomes the space of events on which we may compute statistics. In this case, the identification procedure implies for the constitutive tensors a stochastic character, because they depend on the geometric randomness of protein arrangements. Eventually, it suffices to consider mean, coefficient of variation, skewness, and curtosis to have a reasonable picture of the mechanical behavior described by data distributions produced by numerical simulations. Higher moments than the average allow us to recognize even in the linear constitutive setting a progressive emergence of phenomena that can lead to a critical behavior. Examples of this procedure (although referred to other microstructures) can be found in reference (Gioffrè et al. 2004).
